# Incidence and Predictors of Hospitalization for Bacterial Infection in Community-Based Patients with Type 2 Diabetes: The Fremantle Diabetes Study

**DOI:** 10.1371/journal.pone.0060502

**Published:** 2013-03-25

**Authors:** Emma J. Hamilton, Natalie Martin, Ashley Makepeace, Brett A. Sillars, Wendy A. Davis, Timothy M. E. Davis

**Affiliations:** 1 Department of Endocrinology and Diabetes, Fremantle Hospital, Fremantle, Australia; 2 Department of Geriatric Medicine, Fremantle Hospital, Fremantle, Australia; 3 University of Western Australia, School of Medicine and Pharmacology, Fremantle Hospital, Fremantle, Australia; German Diabetes Center, Leibniz Center for Diabetes Research at Heinrich Heine University Duesseldorf, Germany

## Abstract

**Background:**

The few studies that have examined the relationship between diabetes and bacterial infections have utilized administrative databases and/or have had limited/incomplete data including recognized infection risk factors. The aim of this study was to determine the incidence and associates of bacterial infection severe enough to require hospitalization in well-characterized community-based patients with type 2 diabetes.

**Methods and Findings:**

We studied a cohort of 1,294 patients (mean±SD age 64.1±11.3 years) from the longitudinal observational Fremantle Diabetes Study Phase I (FDS1) and 5,156 age-, gender- and zip-code-matched non-diabetic controls. The main outcome measure was incident hospitalization for bacterial infection as principal diagnosis between 1993 and 2010. We also examined differences in statin use in 52 FDS1 pairs hospitalized with pneumonia (cases) or a contemporaneous non-infection-related cause (controls). During 12.0±5.4 years of follow-up, 251 (19.4%) patients were hospitalized on 368 occasions for infection (23.7/1,000 patient-years). This was more than double the rate in matched controls (incident rate ratio (IRR) (95% CI), 2.13 (1.88–2.42), *P*<0.001). IRRs for pneumonia, cellulitis, and septicemia/bacteremia were 1.86 (1.55–2.21), 2.45 (1.92–3.12), and 2.08 (1.41–3.04), respectively (*P*<0.001). Among the diabetic patients, older age, male sex, prior recent infection-related hospitalization, obesity, albuminuria, retinopathy and Aboriginal ethnicity were baseline variables independently associated with risk of first hospitalization with any infection (*P*≤0.005). After adjustment for these variables, baseline statin treatment was not significant (hazard ratio (95% CI), 0.70 (0.39–1.25), *P* = 0.22). Statin use at hospitalization for pneumonia among the case-control pairs was similar (23.1% vs. 13.5%, *P* = 0.27).

**Conclusions:**

The risk of severe infection is increased among type 2 diabetic patients and is not reduced by statin therapy. There are a number of other easily-accessible sociodemographic and clinical variables that could be used to optimize infection-related education, prevention and management in type 2 diabetes.

## Introduction

A positive association between diabetes and infection was previously the subject of debate in the literature [1]–[3], but recent evidence suggests that bacterial infections are a relatively frequent occurrence in diabetic patients and that there may be an associated increase in morbidity and mortality [4], [5]. Although most studies assessing infections complicating diabetes have been cross-sectional, involved selected (typically hospitalized) patients and/or have not been able to adjust for other important explanatory/confounding variables pertinent to infection risk [5], [6], the weight of evidence suggests that patients with type 2 diabetes have an increased incidence of common community-acquired infections [7], [8], including lower respiratory tract infection, urinary tract infection (UTI), and skin and mucous membrane infections [6]. There is also a substantially increased susceptibility to rare but potentially fatal infections including necrotizing fasciitis and emphysematous pyelonephritis [4], [5]. In addition, diabetes has been identified as an independent risk factor for severe Gram positive blood stream infections [8]–[10], and for hospital-acquired post-operative bacterial infections [11], [12].

Data from observational studies suggest that 3-hydroxy-3-methyl-glutaryl-Coenzyme A reductase inhibitors (statins) may have beneficial effects on the prevention and treatment of bacterial infections in the general population [13]–[15]. However, a recent meta-analysis of randomized placebo-controlled trials found no effect of statin therapy on infection risk or infection-related death [16]. Statin therapy is commonly prescribed for patients with diabetes [17] who are also more susceptible to infection [7], [8], factors that could increase the likelihood of unmasking a beneficial effect of statins on infection risk. There would be difficulties in assessing this validly through a contemporary randomized trial, since there would be a relatively small group of eligible diabetic patients in whom vascular risk was low enough to ethically withhold statin therapy if they were allocated placebo. Observational studies, especially those conducted before evidence of statin cardiovascular benefit became available, are the best source of such comparative data. However, apart from one retrospective UK primary care study in which statin use was associated with a decreased risk of pneumonia [18], there have been no published data regarding the effect of statin therapy on the incidence of infections in diabetic patients.

The aims of the present study were, therefore, to i) identify the incidence and predictors of bacterial infections severe enough to require hospitalization of representative community-based patients with type 2 diabetes, and ii) determine whether statin therapy protects against pneumonia requiring hospitalization in a subset of type 2 patients.

## Methods

### Ethics statement

The FDS1 protocol was approved by the Human Rights Committee at Fremantle Hospital, Fremantle, Western Australia, and all subjects gave written informed consent before participation.

### Patients

The Fremantle Diabetes Study Phase I (FDS1) is a longitudinal observational cohort study of patients from a zip-code-defined urban community of 120,097 people. Descriptions of recruitment, sample characteristics including classification of diabetes type and details of non-recruited patients have been published elsewhere [19], [20]. Of 2,258 diabetic patients identified between April 1993 and June 1996, 1,426 (63%) were recruited to FDS1 and 1,294 had clinically-diagnosed type 2 diabetes. Eligible patients who declined participation were a mean of 1.4 years older than participants, but their sex distribution, the proportion with type 2 diabetes and their use of blood glucose-lowering therapies were similar [19], [20].

### Fremantle Diabetes Study Phase I procedures

Each FDS1 patient underwent comprehensive assessment at entry and was invited to return for similar assessment on an annual basis over a minimum of 5 years [19], [20]. Questionnaire data included demographic, socioeconomic, diabetes-specific and general health data, with ethnic background based on self-selection, country/countries of birth and parents' birth and language(s) spoken at home. Patients provided fasting blood and urine samples for automated biochemical analyses in a single laboratory.

Complications were categorized using standard criteria [19]. A subject was considered as having retinopathy if any grade of retinopathy, including maculopathy, was detected by direct and/or indirect ophthalmoscopy in one or both eyes and/or on more detailed assessment by an ophthalmologist. Peripheral sensory neuropathy was defined as a score of >2/8 on the clinical portion of the Michigan Neuropathy Screening Instrument [21]. Nephropathy was defined as an urinary albumin:creatinine ratio (ACR) >3.0 mg/mmol in a first-morning urine sample. Patients were classified as having ischemic heart disease if there was a self-reported history of, or hospitalization for, myocardial infarction, angina, coronary artery bypass grafting, angioplasty, and/or definite myocardial infarction on a Minnesota-coded electrocardiogram [22]. Self-reported stroke and transient ischemic attack were amalgamated with prior hospitalizations to define baseline cerebrovascular disease status. Peripheral arterial disease (PAD) was considered to be present when the ankle brachial index (ABI) was ≤0.90 or there was a history of any PAD-related lower extremity amputation [23].

Additional endpoint data were obtained from a government register that records details of all deaths and all hospitalizations (whether to a public or private hospital) in the state of Western Australia (WA) and which is part of the larger WA Data Linkage System [24]. These sources provided details of hospital inpatient admissions from the beginning of January 1993 until the end of December 2010.

### Selection of non-diabetic control subjects

It is compulsory for all Australians aged ≥18 years to vote in Federal and State elections and thus all adults resident in the FDS1 catchment area are listed on the electoral roll. Four age-, sex- and zip-code-matched non-diabetic controls were randomly selected from this source for each participant at the time of FDS1 study entry [25]. The requirement for zip-code matching was based on the fact that there are substantial socio-economic and environmental differences between residential districts within the FDS1 catchment area. Non-diabetic status was defined as diabetes not being coded at any time on any WA health database. Matches could not be made for five children and four elderly participants, and these patients were excluded from estimation of incident rate ratios, leaving 1,289 (99.6%) diabetic participants who were matched with 5,156 non-diabetic controls. Five controls died just before entry into FDS1 by their matched case and were therefore excluded. Because the range of data available for the control subjects was limited to date of birth, sex and zip-code, they were used only for estimation of incident rate ratios (IRRs).

### Hospitalization for infection

All hospital admissions with the International Classification of Disease (ICD)-9-CM and ICD-10-AM codes for bacterial infection (including pneumonia, urosepsis, osteomyelitis, cellulitis, meningitis, otitis media and externa, septicemia, bacteremia and abscess; see Table 1) as principal diagnosis were identified. The crude incidence of hospitalization for infection was defined as the number of hospitalizations for infection as principal diagnosis in the two groups of subjects during follow-up divided by the person-years of follow-up from study entry until death or end-December 2010, whichever came first.

**Table 1:** International Classification of Disease (ICD)-9-CM and ICD-10-AM codes for bacterial infection used in the present study.

Infection	ICD-9-CM	ICD-10-AM
Pneumonia	480.1, 480.2 480.8, 480.9, 481, 482.0–.9, 483.0, 485, 486	J12.1, J12.2, J12.8, J12.9, J13, J14, J15.0, J15.1, J15.3, J15.4, J15.5, J15.6, J15.7, J15.8, J15.9, J18.0, J18.8, J18.9
Urosepsis (cystitis, pyelonephritis, prostatitis)	590.1, 590.10, 590.11, 590.2, 595.0, 595.89, 601.0, 601.2, 601.3	N10, N15.1, N30.0, N30.8, N41.0, N41.2, N41.3
Osteomyelitis (acute, chronic, any site, unspecified, periostitis)	730.00–.09, 730.10–.19, 730.20–.29, 730.30–.39	M86.00–.09, M86.10–.19, M86.20–.29, M86.30–.39, M86.40–.49, M86.50–.59, M86.60–.69, M86.80–.89, M86.90–.99
Cellulitis	681.00, 681.10, 681.9, 682	L03.0–.9
Bacterial meningitis	320.0, 320.1, 320.2 320.3, 320.81, 320.82, 320.89, 320.9	G00
Sinusitis, otitis media/externa	380.10, 380.11, 381.00, 381.01, 381.02, 382.00, 382.01, 461.0–.8	H60.0, H60.1, H60.3, H65.0, H65.1, H66.0, J01.0–.9
Septicemia/bacteremia (including meningococcal disease)	036.0, 036.2, 036.3, 036.9, 038.0, 038.1, 038.2, 038.4, 038.40, 038.41, 038.42, 038.9, 041.00, 041.01, 041.02, 041.03, 041.04, 041.05, 041.09, 041.10–.19, 041.3–.7, 041.81, 041.89, 041.9, 790.7	A39.0–.2, A39.4, A39.9, A40, A41.0–.3, A41.51, A41.9, A49
Abscess	324.0, 324.9, 513.0,567.2, 569.5, 572.0	G06, J85.1, J85.2, K63.0, K65.0. K75.0

### Statin use at time of hospitalization for pneumonia

We assessed the effect of statin therapy on community-acquired pneumonia complicating type 2 diabetes because i) it is the most common infection requiring hospitalization, ii) there are relatively objective criteria for its diagnosis including an infiltrate on lung imaging [26], and iii) it is the infection with the most data suggesting the benefits of statins [16], including in diabetes [18]. For the 113 FDS1 patients who were hospitalized with pneumonia up to end-June 2005 (cases), the use of a statin at the time of admission with pneumonia was established by a detailed examination of the patient hospital medical record. Current statin use was also determined, where this was possible, for age and sex-matched FDS1 patients who had been admitted for indications other than infection (controls) within 6 months (mean 0.4 months) either side of the case's pneumonia.

### Statistical methods

The computer package IBM SPSS Statistics 20 (IBM Corporation, Somers, NY, USA) was used. Data are presented as proportions, means (standard deviation, SD), geometric mean (SD range), or, in the case of variables that did not conform to a normal or ln-normal distribution, median [interquartile range, IQR]. For independent samples, two-way comparisons for two proportions were by Fisher's exact test, for normally distributed variables were by Student's *t*-test, and for non-normally distributed variables by Mann-Whitney *U*-test. Cox proportional hazards modelling (forward conditional variable entry and removal with *P*<0.05 and *P*>0.10, respectively) was used to determine independent predictors of time from study entry to first hospitalization for any infection and for the three most common infections (pneumonia, cellulitis, and septicemia/bacteremia) as principal diagnoses. All clinically plausible bivariate variables with *P*<0.20 were considered for entry into the models. McNemar's test was used to determine if there was any statistically significant difference in pre-admission statin use amongst the cases vs controls.

## Results

### Incidence of bacterial infection in diabetic patients and controls

At study entry, the 1,294 FDS1 type 2 participants had a mean±SD age of 64.1±11.3 years, 48.8% were male and their median [IQR] diabetes duration was 4.0 (1.0–9.0) years. Anglo-Celts comprised the main racial/ethnic group (61.5%) with those from Southern European

(17.7%) and other European (8.5%) backgrounds the next largest, followed by Asian (3.3%), Aboriginal (1.5%) and mixed/other (7.5%) origins. There were 15.3% of FDS subjects who were non-fluent in English and 26.0% who had not been educated beyond primary level, with non-Anglo-Celt patients over-represented in these categories. Almost two-thirds (65.8%) of the cohort were married/in a *de facto* relationship.

**Figure 1:**
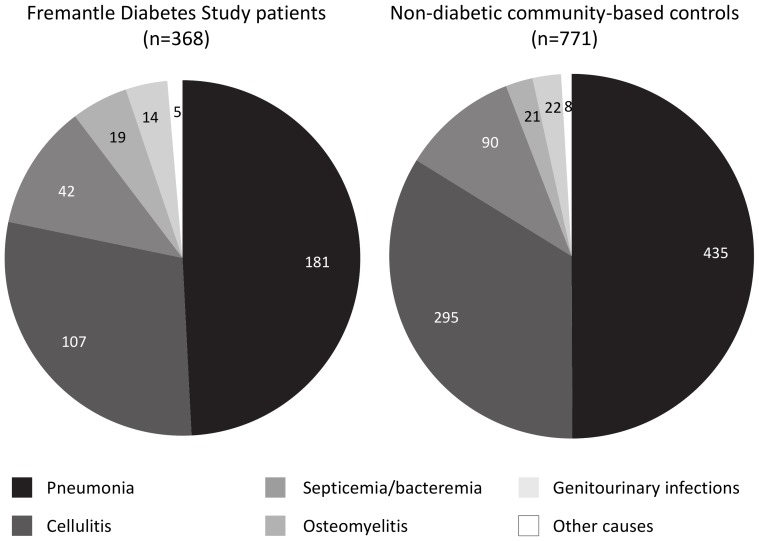
Pie graphs showing the numbers of bacterial infections necessitating hospitalization by type for diabetic patients (left panel) and matched non-diabetic controls (right panel).

During follow-up from study entry until death or 31 December 2010, a total of 15,535 patient-years or a mean±SD of 12.0±5.4 years, 251 (19.4%) were hospitalized on 368 occasions for infection as principal diagnosis. Therefore, the crude incidence (95% CI) of hospitalization for infection was 23.7 (21.3–26.2)/1,000 patient-years. The 368 admissions consisted of pneumonia (n = 181, 49.2%), cellulitis (n = 107, 29.1%), septicemia/bacteremia (n = 42, 11.4%), osteomyelitis (n = 19, 5.2%), genitourinary infection (acute pyelonephritis, renal/perinephric abscess or cystitis; n = 14, 3.8%) and others (meningococcal disease, otitis media, otitis externa, sinusitis and other bacterial infection; n = 5, 1.4%; see Figure 1).

There were 771 hospitalizations for infection in 577 (11.2%) controls over 69,350 patient-years, representing a crude incidence of 11.1 (10.4–11.9)/1,000 patient-years. The 771 admissions comprised pneumonia (n = 435, 56.4%), cellulitis (n = 195, 25.3%), septicemia (n = 90, 11.7%), genitourinary infection (n = 22, 2.9%), osteomyelitis (n = 21, 2.7%) and others (sinusitis, otitis externa and bacterial infection; n = 8, 1.0%; see Figure 1).

The overall incidence of hospitalization for bacterial infections in FDS1 patients with type 2 diabetes was more than double that in the matched controls (IRR (95% CI): 2.13 (1.88–2.42), *P*<0.001). However, there was no significant difference between the proportions of infections by type in the two groups of patients (Chi-squared 8.93, df = 5, *P* = 0.11), and the average number of hospitalizations for infection per patient was also similar (1.47 in the diabetic patients vs 1.34 in the non-diabetic controls). IRRs for pneumonia, cellulitis, and septicemia/bacteremia were, respectively, 1.86 (1.55–2.21), 2.45 (1.92–3.12), and 2.08 (1.41–3.04), all *P*<0.001.

### Bivariate predictors of incident bacterial infections in the diabetic patients

Older age, longer diabetes duration, higher BMI, systolic blood pressure, fasting serum triglycerides and urinary albumin:creatinine ratio (ACR), ischemic heart disease, retinopathy, peripheral neuropathy and eGFR <60 ml/min/1.73 m^2^, and prior hospitalization for any infection (as principal diagnosis between January 1982 and FDS1 study entry) were all associated with hospitalization for any infection during follow-up in bivariate analyses (*P*<0.05; see Table 2). There was no significant difference in fasting plasma glucose or HbA_1c_ at baseline between the two groups (*P*≥0.07). In addition, there was no association between incident infection and serum total or HDL-cholesterol concentrations or use of lipid-lowering agents including statins (*P*≥0.21).

**Table 2:** Bivariate baseline associates of hospitalization for any infection after study entry.

	*No hospitalization*	Hospitalization	*P*-value
Number (%)	1043 (80.6)	251 (19.4)	
Age (years)	63.6±11.4	66.1±10.6	0.001
Male (%)	47.6	53.8	0.08
Age at diabetes diagnosis (years)	57.7±11.6	58.9±11.5	0.13
Diabetes duration (years)	4.0 [0.9–8.0]	4.1 [1.5–11.0]	0.012
Body mass index (kg/m^2^)	29.4±5.4	30.3±5.6	0.012
Ethnic background: Anglo-Celt	61.5	61.8	0.14
Southern European	17.4	19.1	
Other European	8.2	9.6	
Asian	3.8	1.2	
Mixed/other	7.9	6.0	
Aboriginal	1.2	2.4	
Fasting serum glucose (mmol/L)	8.4 [6.8–10.7]	8.6 [7.1–11.3]	0.16
HbA_1c_ (%)	7.4 [6.4–8.7]	7.6 [6.5–9.1]	0.07
Diabetes treatment: Diet	32.9	28.0	0.30
Oral agents	55.4	58.8	
Insulin ± oral agents	11.7	13.2	
Systolic blood pressure (mmHg)	150±24	155±23	<0.001
Diastolic blood pressure (mmHg)	80±11	81±11	0.53
On blood pressure-lowering medication (%)	49.9	55.8	0.11
Total serum cholesterol (mmol/L)	5.5±1.1	5.5±1.0	0.45
Serum HDL-cholesterol (mmol/L)	1.07±0.32	1.04±0.34	0.21
Serum triglycerides (mmol/L)	1.9 (1.1–3.2)	2.0 (1.2–3.5)	0.016
On lipid-lowering medication (%)	11.1	8.0	0.17
Taking statin therapy (%)	6.9	6.0	0.68
Taking ≥75 mg/day aspirin (%)	20.8	25.6	0.11
Urinary albumin:creatinine (mg/mmol)	3.0 (0.7–12.6)	3.8 (0.8–17.9)	0.012
Estimated glomerular filtration rate <60 mL/min/1.73 m^2^ (%)	41.4	51.8	0.003
Any retinopathy (%)	15.1	22.1	0.011
Peripheral neuropathy (%)	29.4	37.0	0.028
Peripheral arterial disease (%)	29.1	30.2	0.76
Cerebrovascular disease (%)	9.3	12.7	0.10
Ischemic heart disease (%)	28.3	35.1	0.038
Any exercise in past two weeks (%)	73.1	67.5	0.08
Smoking status (%): Never	46.2	38.4	0.07
Ex-smoker	38.9	46.0	
Current	14.9	15.6	
Alcohol use (average standard drinks/day)	0 [0–0.3]	0 [0–0.8]	0.12
Prior hospitalization for any infection (%)*	5.6	10.4	0.010

Data are %, mean ± SD, median [IQR] or geometric mean (SD range);*between January 1982 and study entry

### Independent predictors of time to first incident infection in the diabetic patients

In a Cox proportional hazards model (see Table 3), older age, male sex, higher BMI, higher urine ACR, retinopathy, Aboriginal racial background, and prior hospitalization for any infection (as principal diagnosis between January 1982 and FDS1 study entry) all increased the risk of hospitalization with any infection during follow-up (all *P*≤0.006). After adjusting for these variables, statin therapy was not protective against hospitalization for any infection (hazard ratio (95% CI) 0.70 (0.39–1.25), *P* = 0.22).

**Table 3:** Cox proportional hazards modelling showing hazard ratios (HR) and 95% CI for independent baseline associates of time to first hospitalization for i) any infection, ii) pneumonia, iii) cellulitis, and iv) septicemia/bacteremia as principal diagnosis.

	HR (95% CI)	P-value
i) Any infection (n = 251)		
Age (increase of 10 years)	1.71 (1.47–1.99)	<0.001
Male	1.59 (1.22–2.07)	0.001
BMI (increase of 1 kg/m^2^)	1.04 (1.02–1.07)	0.001
Log_e_ (urinary albumin:creatinine ratio (mg/mmol))*	1.15 (1.06–1.26)	0.002
Retinopathy	1.57 (1.14–2.18)	0.006
Aboriginal	3.22 (1.40–7.38)	0.006
Prior hospitalization for any infection	2.76 (1.77–4.31)	<0.001
ii) Pneumonia (n = 140)		
Systolic blood pressure (increase of 1 mmHg)	1.015 (1.01–1.023)	<0.001
Log_e_ (serum triglycerides (mmol/L))*	0.67 (0.48–0.93)	0.015
Ischemic heart disease history	1.78 (1.25–2.55)	0.001
Aboriginal racial background	8.35 (2.81–24.8)	<0.001
Prior hospitalization for any infection	2.00 (1.12–3.56)	0.019
iii) Cellulitis (n = 107)		
Age (increase of 10 years)	1.37 (1.07–1.77)	0.014
Peripheral neuropathy	1.78 (1.05–3.00)	0.032
Prior hospitalization for any infection	5.14 (2.46–10.7)	<0.001
iv) Septicemia/bacteremia (n = 42)		
Fasting serum glucose (increase of 1 mmol/L)	1.22 (1.08–1.38)	0.001
Retinopathy present	3.03 (1.31–7.04)	0.010

* A 2.72-fold increase in serum triglycerides or urinary albumin:creatinine ratio corresponds to an increase of 1 in ln(triglycerides) or ln(ACR), respectively.

Significant independent associates of specific infections (see Table 3) comprised higher systolic blood pressure, lower serum triglycerides, known ischemic heart disease, Aboriginal racial background and prior infection-related hospitalization in the case of pneumonia; older age, peripheral neuropathy and prior hospitalization for any infection for cellulitis; and fasting serum glucose and retinopathy for septicemia/bacteremia.

### Effect of statin use on hospitalization for pneumonia in diabetic patients

We were able to identify a subset of 52 case-control pairs of FDS1 participants in whom statin use was confirmed for pneumonia-related or closely contemporaneous non-infection-related admissions in cases and controls, respectively. Coding for pneumonia was confirmed by chart review in all the cases. There was no significant difference in the proportion of patients using statin therapy amongst FDS1 patients admitted with pneumonia compared to those hospitalized for indications other than infection (23.1% vs 13.5%, *P* = 0.27).

## Discussion

The present study shows that type 2 diabetes is associated with a more than two-fold increase in the rate of hospitalization for any bacterial infection in representative patients from an urban community setting. One in five FDS1 type 2 patients was admitted with infection as a primary diagnosis during an average follow-up of 12 years compared with one in nine of the matched non-diabetic control subjects drawn from the same population over the same period. The distribution of the type of bacterial infection was similar in the two groups. Community-acquired pneumonia was the most common, accounting for approximately half of all admissions, but cellulitis, septicemia/bacteremia, osteomyelitis and genitourinary infections were also prominent causes. In the diabetic patients, independent associates of hospitalization with bacterial infection included older age, male sex, an infection-related admission before recruitment to FDS1, obesity, microangiopathy (retinopathy and albuminuria) and Aboriginal racial origin, but our data provide no evidence that statin therapy helps prevent hospital admission with infection, including pneumonia, in patients with type 2 diabetes.

The IRR for hospitalization for any infection in our study (2.13) was similar to the relative risk of 2.17 for the same outcome in a large retrospective Canadian administrative database study of patients with diabetes of unspecified type and matched non-diabetic controls [4]. The distribution by type of bacterial infection was not significantly different between our FDS1 participants and the matched non-diabetic controls, consistent with the three most common infections also being associated with an approximate doubling of the risk of hospitalization (with an IRR between 1.86 and 2.45 for pneumonia, cellulitis, and septicemia/bacteremia). Available published data support this finding. In a Dutch prospective general practice study, the adjusted odds ratios for medical attendances for the major specific types of infection in patients with type 2 diabetes were all increased by ≥32% compared to control patients who had hypertension without diabetes [6]. For pneumonia, the relative risk of hospitalization was increased by ≥23% in Danish diabetic patients [27], [28], while studies from the US [29] and UK [30] have shown a diabetes-associated relative risk for UTI of 2.1 to 2.2.

There are a variety of mechanisms by which diabetic patients are at increased risk of bacterial infection. These include hyperglycemia-related impairment of immune function and the adverse effects of the vascular and neuropathic complications of diabetes on tissue structure and function [5]. The relationship between glycemic control and infection in previous studies has been strongest for skin infections and periodontal disease and relatively weak in the case of respiratory tract and genitourinary infections [5]. We found no association between HbA_1c_ at FDS1 enrolment and subsequent risk of any or site-specific infections in our cohort. However, microangiopathy (retinopathy, neuropathy and/or nephropathy) and/or ischemic heart disease were independently associated with increased risk of hospitalization for any bacterial infection and the three most common sub-types, and may be surrogates for chronic glycemic exposure with adverse effects on immune and other tissues. This latter effect might involve the lung in the case of pneumonia [31], the integrity of skin, soft tissue and skeleton in cases of cellulitis (as evidenced by the association with neuropathy in the present study), osteomyelitis and septicemia/bacteremia [32], [33], and bladder function in genitourinary infections [34], [35].

Age, male sex and Aboriginal racial background were independently associated with incident hospitalization with any infection in the FDS1 patients. Community-acquired infections such as pneumonia [36] and cellulitis (as also in the present study) [37] are more common in older age groups in the general population and our data suggest that this is also the case in type 2 diabetes. Although UTI complicating type 2 diabetes is much more common amongst females than males [38], [39], there is evidence of a male excess in recent general population studies of other infections [40]–[44] which is likely to apply to diabetes and account for the significant sex difference in risk of hospitalization with any bacterial infection observed in the FDS1 cohort.

The more than three-fold increase in risk of any infection and of pneumonia in particular in Aboriginal vs non-Aboriginal patients reflects previous studies from WA and other Australian states that have shown an increased rate of hospitalization for respiratory infections and pneumonia amongst Aboriginal groups [45], [46]. In addition, infection has been reported to be the primary reason for hospitalization in 60% of central Australian Aboriginal patients with diabetes, most commonly foot infections [47]. There are several proposed explanations for the increased rate of respiratory and other infections among Aboriginal patients including overcrowding, social disadvantage, poor living conditions, and limited access to care, education and resources to assist with management of diabetes and other chronic health problems such as high smoking rates [45], [47].

A high BMI was independently associated with incident hospitalization with any infection in our diabetic patients. Obesity is known to have adverse effects on immune function and to increase susceptibility to infections such as pneumonia [48], a relationship that was independent of other diabetes-related variables such as HbA_1c_ and vascular complications in our cohort. The association between systolic blood pressure at FDS1 entry and subsequent hospitalization with community-acquired pneumonia is likely to reflect the strengthening association between chronic cardiac disease associated with hypertension such as heart failure and pneumonia observed in population-based studies [49], [50]. Since triglyceride-rich lipoproteins are hypothesized to be a component of the innate host immune response to bacterial infections [51], the inverse association between fasting serum triglycerides and incident pneumonia could mean that this contribution to immunity is attenuated in patients with relatively low triglyceride levels.

The associations between recent prior infection-related hospitalization and any incident infection as well as pneumonia and cellulitis are consistent with the observation that patients with diabetes are at increased risk of recurrent community-acquired infections [7]. An alternative explanation is that some diabetic patients, including those with a heavy burden of complications and co-morbidities, are vulnerable to recurrent severe infections.

Statins are frequently used for lipid-lowering in patients with diabetes but also have modulatory effects on innate and adaptive immune systems and anti-inflammatory effects, and may help to counteract undesirable effects of sepsis on the coagulation system [13]. There is some evidence suggesting that statins may have a beneficial role in the prevention and treatment of infection [13]–[15], but this was not confirmed by a meta-analysis of randomized placebo-controlled trials of statin therapy [16]. The only published study to have demonstrated a lower risk of pneumonia in adult diabetic patients treated with statins used the United Kingdom General Practice Research Database [18]. Only a very small percentage of patients (1.9%) were taking statin therapy at the time (between 1987 and 2001), the diagnosis of pneumonia was made by the general practitioner at first attendance, and there were very few confounding variables available for incorporation in multivariate analysis. We found statin therapy was not protective for hospitalization for infection amongst our cohort of well-characterized community-based patients with type 2 diabetes, which is consistent with the evidence from the general population meta-analysis [16].

Our study had limitations. First, the observed differences in IRR between diabetic and non-diabetic samples may have been due, in part, to between-group differences in healthcare-seeking behaviour and/or the treating physician's threshold for hospitalization. Perceptions of diabetes as an important co-morbidity may have made infection-related admission of FDS patients more likely compared to those of matched controls. However, as with other such studies [4], this seems unlikely from the consistency of the data across types of infection and the fact that those for which hospitalization is less discretionary such as septicaemia or bacteremia also had an increased diabetes-associated risk. Second, we did not have access to data other than age, gender and postcode for the matched controls and so could not adjust IRRs for between-group differences in other variables (such as obesity and ethnicity) that might have impacted on the risk of infection. Third, we did not have complete data on prior vaccination for either the FDS whole cohort or patients in the statin case-control pneumonia study. However, in a separate FDS sub-study [7], type 2 diabetic participants were at least as likely as their non-diabetic spouses to have received influenza vaccine within the past year and pneumococcal vaccine within the previous 5 years. Fourth, it is likely that variables such as statin use and glycemic control changed during the course of the study with consequences for infection risk, hospitalization and mortality. However, in the subset of patients in whom statin use was confirmed at the time of hospitalization by review of the medical record, this also did not identify a significant difference in statin use amongst patients admitted with pneumonia compared to those admitted for non-infectious indications. The strengths of the present study include the prospective design, large patient numbers, detailed baseline assessment and capture of endpoints through a validated data linkage system.

In summary, the finding that patients with diabetes in our FDS1 cohort had double the incidence of hospitalization for infectious diseases vs that of matched non-diabetic controls is consistent with data from other sources including a large administrative database study [4]. Older age, male gender, Aboriginal racial background, BMI and chronic vascular complications were independent associates of the serious bacterial infections requiring hospitalization in our diabetic patients. All these are easily accessible variables that could be used to target patients at increased risk of serious infections with education and counselling regarding preventative measures such as vaccination and foot care, as well as prompting early intensive management of infections with the potential to become severe. Statins are a group of drugs with clear evidence for cardiovascular benefit in type 2 diabetes but we found no evidence that they altered the risk of serious infections.
